# Erythrocyte Membrane Fatty Acid Remodeling and Zinc Protoporphyrin Alterations Associated with Anemia Severity in Hemodialysis Patients

**DOI:** 10.3390/biomedicines14071582

**Published:** 2026-07-15

**Authors:** Merve Kılıç, Hamad Dheir, Mahmud İslam, Zafer Ercan, Muhittin Abdulkadir Serdar

**Affiliations:** 1 Department of Anesthesia Program, Advanced Vocational School, Dogus University, 34775 Istanbul, Turkey; 2 Department of Nephrology, Sakarya University Faculty of Medicine, 54187 Sakarya, Turkey; hamaddheir@sakarya.edu.tr (H.D.); mislam@sakarya.edu.tr (M.İ.); 3 Department of Nephrology, Sakarya University Training and Research Hospital, 54100 Sakarya, Turkey; zafercan7@gmail.com; 4 Department of Medical Biochemistry, School of Medicine, Acibadem Mehmet Ali Aydinlar University, 34752 Istanbul, Turkey

**Keywords:** erythrocyte membrane fatty acids, hemodialysis patients, hemoglobin, ZnPP, iron-restricted erythropoiesis

## Abstract

**Background**: This study investigated the relationship between erythrocyte membrane fatty acids, omega-3 index, and zinc protoporphyrin (ZnPP) levels in hemodialysis (HD) patients across hemoglobin-defined anemia severities. **Methods**: This prospective cross-sectional study included 117 chronic HD patients and 28 healthy controls. ZnPP was measured by high-performance liquid chromatography, and erythrocyte membrane fatty acids by gas chromatography–mass spectrometry. **Results**: HD patients showed a significantly lower omega-3 index than controls (*p* < 0.0001), although it did not differ across hemoglobin-defined groups (*p* = 0.589). Palmitic and erucic acid were higher, whereas oleic acid was lower, in patients with lower hemoglobin. In unadjusted analyses, hemoglobin correlated inversely with palmitic acid (r = −0.220, *p* = 0.017) and erucic acid (r = −0.183, *p* = 0.048), whereas the positive association with oleic acid (r = 0.451, *p* < 0.0001) remained significant after false discovery rate (FDR) correction. ZnPP increased as hemoglobin decreased (*p* = 0.013) and was negatively associated with hemoglobin (r = −0.406, *p* < 0.0001). **Conclusions**: HD patients with lower hemoglobin showed altered erythrocyte membrane fatty acid composition; among fatty acids, only the oleic acid–hemoglobin association remained significant after FDR correction. ZnPP rose as hemoglobin declined and was independently inversely associated with hemoglobin after multivariable adjustment (β = −0.051, *p* < 0.0001), independent of any dichotomous reference definition, supporting ZnPP as a continuous marker of anemia severity and iron-restricted erythropoiesis in HD patients.

## 1. Introduction

Chronic kidney disease (CKD) is associated with progressive impairment of erythropoiesis due to decreased erythropoietin (EPO) production, chronic inflammation, oxidative stress, and disturbances in iron metabolism, ultimately resulting in anemia, one of the most common complications of CKD [1]. According to KDIGO 2026 Clinical Practice Guideline for Anemia in CKD, anemia is defined as Hb < 13 g/dL in men and <12 g/dL in women [2] and its prevalence increases with CKD progression [3]. Hemodialysis (HD) patients are particularly susceptible to iron deficiency because of repetitive blood loss during dialysis procedures, impaired gastrointestinal iron absorption, inflammation-mediated iron sequestration, and increased iron demand during erythropoiesis. Approximately 2 g of iron is lost annually [4]. In the present study, iron status was characterized using KDIGO-based iron indices (Transferrin saturation (TSAT) and ferritin), interpreted together with hemoglobin levels and overall clinical assessment to exclude alternative causes of anemia [5]. For the analysis comparing laboratory and membrane parameters, patients were stratified according to hemoglobin levels rather than a fixed iron-deficiency cut-off, allowing evaluation of how these parameters change as anemia severity increases. Notably, in HD patients, iron indices frequently reflect iron-restricted erythropoiesis (elevated ferritin with low transferrin saturation) rather than absolute iron deficiency, owing to chronic inflammation [6].

Zinc protoporphyrin (ZnPP) serves as a sensitive indicator of iron-restricted erythropoiesis, reflecting impaired incorporation of iron into protoporphyrin during the final step of heme synthesis. Under iron-deficient conditions, zinc competitively replaces iron in the protoporphyrin ring, resulting in increased ZnPP formation. Unlike ferritin, ZnPP is considered less affected by inflammatory status and may therefore provide additional diagnostic value in distinguishing iron deficiency in HD patients. Biomarkers such as Hb, ferritin, soluble transferrin receptor, and ZnPP are commonly used to evaluate iron status and iron-restricted erythropoiesis in patients with chronic kidney disease. Since ferritin is an acute-phase reactant and may be elevated in inflammation, inflammation-resistant markers like ZnPP are valuable [7,8].

In addition to hematological disturbances, CKD is characterized by profound alterations in lipid metabolism and erythrocyte membrane composition. Previous studies demonstrated that HD patients exhibit abnormal erythrocyte membrane fatty acid profiles associated with oxidative stress, inflammation, impaired membrane fluidity, and cardiovascular risk [9,10]. Omega-3 and omega-6 polyunsaturated fatty acids (PUFAs) affect membrane structure, inflammation, and gene regulation [11,12]. Omega-3 is thought to reduce cardiovascular risk and may offer protective effects in end-stage kidney disease [13]. Moreover, saturated fatty acids may contribute to inflammation and oxidative injury, whereas monounsaturated and polyunsaturated fatty acids may exert protective effects on erythrocyte stability and cellular metabolism.

Despite increasing evidence regarding erythrocyte membrane lipid alterations in CKD, studies specifically evaluating the relationship between fatty acid composition and anemia severity in HD patients remain limited. We hypothesized that HD patients with lower hemoglobin levels exhibit a distinct erythrocyte membrane fatty acid signature characterized by reduced omega-3 index, elevated saturated fatty acids, and increased ZnPP levels. Therefore, this study aimed to (i) compare omega-3 index and erythrocyte membrane fatty acid profiles between HD patients and healthy controls; (ii) evaluate associations between fatty acid composition and anemia-related biochemical markers across hemoglobin-defined groups; and (iii) investigate the potential utility of ZnPP as a biomarker associated with declining hemoglobin and iron-restricted erythropoiesis in HD patients.

## 2. Materials and Methods

### 2.1. Study Population

This prospective, non-interventional, cross-sectional clinical study included 117 chronic HD patients and 28 healthy controls recruited at Sakarya University Training and Research Hospital between 1 September 2023 and 1 December 2023. Patient recruitment was performed at Sakarya University Training and Research Hospital, whereas laboratory analyses and experimental procedures were conducted at Acıbadem Mehmet Ali Aydınlar University. Healthy controls consisted of 28 individuals without known renal or inflammatory disease (mean age 55.8 ± 18.3 years; 53.6% females). No significant differences were observed between healthy controls and HD patients with respect to age (55.8 ± 18.3 vs. 61.4 ± 16.8 years, *p* = 0.149) or sex distribution (42.7% vs. 53.6% females, *p* = 0.410). Ethical approval was obtained from Acıbadem Mehmet Ali Aydınlar University ATADEK (Decision No: 2023-10/340, dated 16 June 2023). Additional informed consent was obtained from all individual participants for whom identifying information is included in this article. Inclusion criteria were age ≥ 18, CKD diagnosis, HD treatment for ≥90 days (3 days/week), and informed consent. The ≥90-day criterion was applied to ensure inclusion of patients receiving stable maintenance hemodialysis and to minimize the influence of acute metabolic and hematological changes occurring during the initiation phase of dialysis treatment. Exclusion criteria included pregnancy, breastfeeding, impaired consciousness, infection, malignancy, liver or hematologic disorders, bleeding, anticoagulant use, malabsorption, or participation in other studies.

### 2.2. Collection and Storage of Analysis Samples

Blood samples were collected into tubes containing 15 mg/mL EDTA at the Nephrology Clinic of Sakarya Education and Research Hospital after 12 h of fasting before hemodialysis. Measurements were made in the Biochemistry Laboratory of the same hospital using BC-6200 and BC-6000 hematology analyzers (Mindray Bio-Medical Electronics Co., Shenzhen, China) after routine daily quality control. Serum was obtained by centrifuging the samples at 3000 rpm for 10 min using a Hettich Universal 320R centrifuge (Andreas Hettich GmbH & Co. KG, Tuttlingen, Germany), and all samples other than daily analysis were stored at −80 °C.

### 2.3. Protocol for ZnPP Measurement by HPLC

Zinc protoporphyrin (ZnPP), protoporphyrin, and mesoporphyrin are light-sensitive compounds; therefore, sample exposure to light was minimized throughout the procedure. The sample preparation procedure was adapted from Hart et al. [14]. Briefly, 100 µL of whole blood collected in EDTA tubes or external standards was transferred into test tubes. Then, 10 µL of internal standard solution containing mesoporphyrin (1 mg mesoporphyrin dissolved in 10 mL dimethyl sulfoxide [DMSO]) and 300 µL of extraction buffer composed of ethyl acetate/acetic acid (4:1, *v*/*v*) were added. Tubes were centrifuged at 10,000× *g* for 10 min, and the supernatant was collected. A 20 µL aliquot of the collected supernatant was injected into the HPLC system.

ZnPP analysis was performed using a Thermo Scientific Dionex UltiMate 3000 FLD-3000 system (Thermo Fisher Scientific, Waltham, MA, USA). Chromatographic separation was achieved using a Hypersil GOLD reversed-phase column (250 mm × 4.6 mm, 5 µm particle size; Thermo Fisher Scientific, Waltham, MA, USA) equipped with a guard column. The mobile phase consisted of methanol and ammonium acetate buffer (76:24, *v*/*v*; pH 7.2), delivered at a flow rate of 1.4 mL/min. The column temperature was maintained at 33 °C. Fluorescence detection was performed at an excitation wavelength of 420 nm and an emission wavelength of 588 nm. Detailed assay validation parameters specific to the current study were not separately evaluated, as the analytical protocol was adapted from a previously validated method [14].

### 2.4. The Protocol for Measuring Erythrocyte Membrane Fatty Acids by GC-MS

For GC-MS analysis, erythrocyte samples were derivatized using boron trifluoride-methanol and hexane to generate fatty acid methyl esters (FAMEs). Briefly, 50 µL RBC samples were mixed with 250 µL of 14% boron trifluoride-methanol and 250 µL hexane, flushed with argon gas, and heated at 100 °C for 10 min. After cooling, 2 mL isooctane containing 0.001% butylated hydroxytoluene (BHT) was added, and the upper organic phase was collected for analysis.

Fatty acid methyl esters were analyzed using a Thermo Scientific TRACE 1300 Series Gas Chromatography system coupled with an ISQ LT Quadrupole Mass Spectrometer (Thermo Fisher Scientific, Waltham, MA, USA). Chromatographic separation was achieved using a TraceGOLD TG-5SilMS GC column (30 m × 0.25 mm × 0.25 µm) with hydrogen as the carrier gas at a constant flow rate of 1.6 mL/min. The oven temperature program was initiated at 80 °C, gradually increased to 250 °C, and maintained to ensure adequate sterol separation.

Fatty acids were identified by comparing retention times and mass spectra with standard fatty acid mixtures. Method validation demonstrated acceptable analytical performance with linearity between 2.5–1000 µg/mL (r^2^ = 0.986–0.9951), recovery rates of 85–108%, LOD values between 0.27–4.5 µg/mL, and repeatability ranging from 88–96%.

External standards and internal quality control samples were analyzed periodically throughout the study to ensure analytical stability and reproducibility.

### 2.5. Statistical Analysis

Statistical analyses were conducted using IBM SPSS Statistics Version 29.0 (IBM Corp., Armonk, NY, USA). Data distribution was assessed using the Shapiro–Wilk and Kolmogorov–Smirnov tests. Depending on data distribution, appropriate parametric (ANOVA and Student’s *t*-test) or non-parametric (Kruskal–Wallis, Mann–Whitney U, and Spearman correlation) tests were applied. Statistical significance was defined as *p* < 0.05. Continuous variables were expressed as mean ± standard deviation (SD) or median (interquartile range, IQR), whereas categorical variables were presented as percentages.

The final sample of 117 HD patients and 28 healthy controls is consistent with sample sizes reported in comparable cross-sectional studies evaluating erythrocyte membrane fatty acid profiles in HD patients [13,15].

To account for the large number of correlation analyses and to limit the risk of type I error, the Benjamini–Hochberg false discovery rate (FDR) procedure was applied across the complete correlation matrix, and adjusted q-values were calculated, An FDR-adjusted q < 0.05 was considered statistically significant. The Benjamini–Hochberg method was preferred over the Bonferroni correction because the tests were numerous and mutually correlated, rendering Bonferroni overly conservative. Associations that were significant in unadjusted analyses but did not remain significant after FDR correction were interpreted as exploratory and hypothesis-generating.

To determine whether the biomarkers of interest were independently associated with hemoglobin after accounting for potential confounders, a multivariable linear regression analysis was performed with hemoglobin as the dependent variable. Palmitic acid, oleic acid, erucic acid, and ZnPP were entered as independent variables, with adjustment for age, sex, diabetes mellitus, C-reactive protein (CRP), iron supplementation, and EPO therapy. Iron status indicators (ferritin, transferrin saturation, and serum iron) were not included as covariates, as they lie on the causal pathway between the studied biomarkers and hemoglobin and would introduce overadjustment. Regression results were reported as unstandardized regression coefficients (β) with 95% confidence intervals (CIs) and corresponding *p*-values.

Receiver operating characteristic (ROC) curve analysis was performed as a secondary analysis to evaluate the diagnostic performance of ZnPP and conventional hematological and iron-related parameters for identifying iron deficiency anemia (IDA). IDA was operationally defined as anemia together with transferrin saturation (TSAT) < 20% and ferritin < 200 ng/mL. Based on these criteria, 43 patients were classified as IDA and 74 patients as non-IDA. The area under the curve (AUC) was reported with its 95% confidence interval (CI), and the statistical significance of each AUC was tested against the null value of 0.5. For each biomarker, the optimal cut-off value was determined using the Youden index, and the corresponding sensitivity, specificity, positive likelihood ratio (LR+), and negative likelihood ratio (LR−) were calculated.

## 3. Results

### 3.1. Demographic Findings of Hemodialysis Patients

The demographic and clinical characteristics of the study population are summarized in Table 1.

The most common underlying causes of CKD were hypertension (*n* = 66, 56.4%), diabetes mellitus (*n* = 35, 29.9%), and urological disorders (*n* = 8, 6.8%). Some patients had multiple contributing etiologies; therefore, percentages may exceed 100%. Dialysis duration was 3–12 months in 20 patients (17.1%), 13–60 months in 59 patients (50.4%), and >61 months in 38 patients (32.5%). Fifteen patients (12.8%) were smokers, and three (2.6%) reported alcohol use. Vascular access consisted of arteriovenous fistula in 63 patients (53.8%) and indwelling catheter in 54 patients (46.2%). During follow-up, 45 patients (38.5%) received iron supplementation, 75 (64.1%) received erythropoietin (EPO), and 9 (7.7%) were treated with ACE inhibitors/angiotensin II receptor blockers (ACE-I/ARB).

Healthy controls had a mean age of 55.8 ± 18.3 years and consisted of 53.6% females and 46.4% males. No statistically significant differences were observed between healthy controls and HD patients with respect to age (55.8 ± 18.3 vs. 61.4 ± 16.8 years, *p* = 0.149) or sex distribution (53.6% vs. 42.7% females, *p* = 0.410).

### 3.2. Erythrocyte Membrane Fatty Acid Levels of Patients

A total of 145 individuals, comprising 117 patients with chronic kidney disease and 28 healthy controls, participated in the erythrocyte membrane fatty acid study. The fatty acid levels in the erythrocyte membranes are presented in Table 2.

Differences in fatty acids between the patient and control groups were tested with the Mann–Whitney U test. Accordingly, the Omega-3 index was lower in HD patients than in the control group (*p* < 0.0001). Statistically significant differences were found between the patient and control groups in erythrocyte membrane fatty acids including palmitic acid (*p* = 0.001), stearic acid (*p* < 0.001), EPA (*p* < 0.0001), DHA (*p* = 0.010), erucic acid (*p* = 0.030), behenic acid (*p* = 0.045), total cholesterol (*p* < 0.0001), HDL cholesterol (*p* < 0.0001) and LDL cholesterol (*p* < 0.0001).

Correlations between erythrocyte membrane fatty acids and biochemical parameters are presented in Table 3. Regarding saturated fatty acids, palmitic acid showed weak inverse correlations with RBC (r = −0.187, *p* = 0.044), Hb (r = −0.220, *p* = 0.017), and HCT (r = −0.197, *p* = 0.033) in unadjusted analyses. However, these associations did not remain statistically significant after Benjamini–Hochberg FDR correction and should therefore be interpreted as exploratory.

In contrast, oleic acid, a monounsaturated fatty acid, showed strong positive associations with RBC (r = 0.442), Hb (r = 0.451), and HCT (r = 0.466) (all *p* < 0.001), and these associations remained significant after FDR correction, suggesting a potential role in erythropoietic activity.

Among long-chain and very-long-chain fatty acids, erucic acid showed weak inverse correlations with RBC (r = −0.226, *p* = 0.014), Hb (r = −0.183, *p* = 0.048), and HCT (r = −0.239, *p* = 0.009) in unadjusted analyses. However, these associations did not remain significant after FDR correction and should therefore be considered exploratory. Similar findings were observed for 7,10,13-eicosatrienoic acid and arachidonic acid, whose correlations did not remain significant after adjustment for multiple testing.

Additional weak correlations observed in the unadjusted analyses did not remain statistically significant after FDR correction and were therefore not further interpreted as confirmatory findings.

### 3.3. Patients’ Hemoglobin Levels and the Effect of These Levels on Their Laboratory Values

This study included 117 HD patients, and the relationship between hemoglobin-defined groups and laboratory parameters is presented in Table 4. Patients were stratified into four hemoglobin-defined groups according to clinically relevant hemoglobin ranges observed within the study cohort: Group 1 (13.0–14.3 g/dL, *n* = 12), Group 2 (11.0–12.7 g/dL, *n* = 38), Group 3 (9.0–10.9 g/dL, *n* = 50), and Group 4 (6.3–8.9 g/dL, *n* = 17). The group boundaries were selected to reflect progressively lower hemoglobin levels while maintaining clinically meaningful categories and adequate numbers of patients within each group. Group boundaries were defined a priori on the basis of clinically meaningful hemoglobin intervals rather than statistical quantiles; because the cohort contained no patient with a hemoglobin value of 12.8–12.9 g/dL, no observations were excluded by these cut-points.

Statistically significant differences were observed between Hb groups for Hb, RBC, HCT, ZnPP, ferritin, albumin, palmitic acid, oleic acid, and erucic acid levels (all *p* < 0.05) (Table 4).

**Figure 1:**
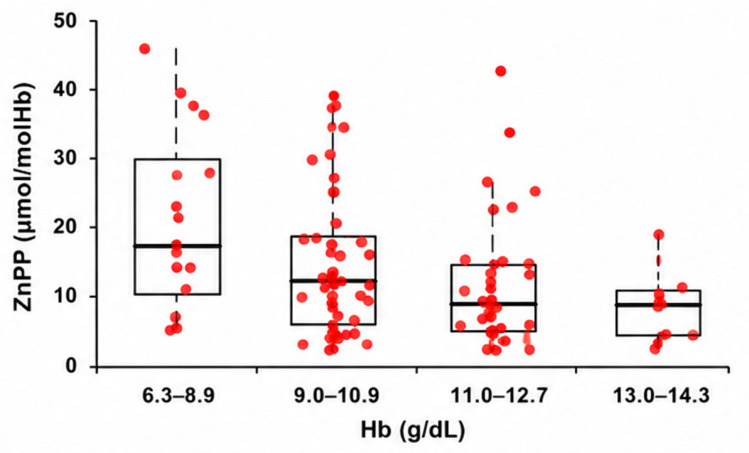
Distribution of ZnPP (μmol/molHb) according to different hemoglobin levels.

A statistically significant difference was found between the Hb groups in terms of ZnPP levels (*p* = 0.013). In post hoc pairwise testing, the only statistically significant difference was between Group 4 and Group 3 (Table 4). ZnPP concentrations were highest in Group 4, although the pattern was not strictly monotonic across all hemoglobin-defined groups (Figure 1). In addition, ZnPP levels were negatively correlated with hemoglobin levels (r = −0.406, *p* < 0.0001). A negative correlation was observed between hemoglobin concentration and ZnPP levels, indicating that ZnPP concentrations tended to increase as hemoglobin levels decreased. These findings suggest that ZnPP may serve as a useful marker of iron-restricted erythropoiesis in hemodialysis patients.

**Figure 2:**
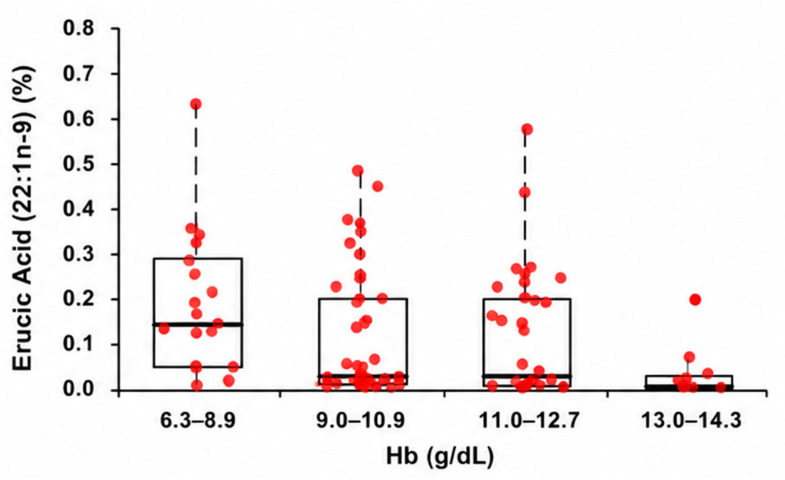
Distribution of erucic acid (22:1n-9) percentages according to different hemoglobin levels.

**Figure 3:**
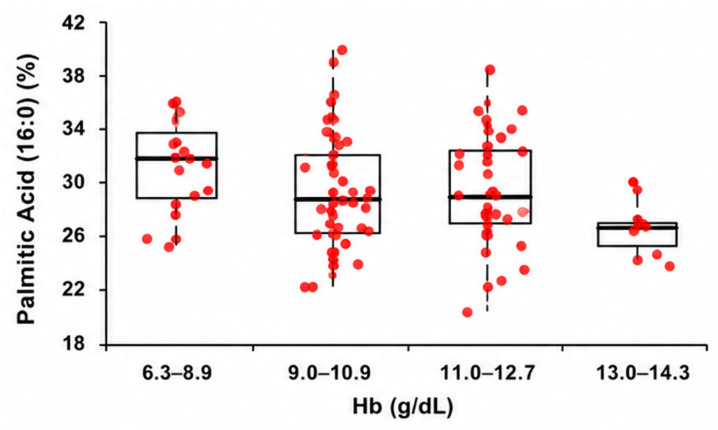
Distribution of palmitic acid (16:0) percentages according to different hemoglobin levels.

An examination of fatty acids revealed statistically significant differences between Hb groups in terms of erucic acid (*p* = 0.004) (Figure 2) and palmitic acid (*p* = 0.016) (Figure 3). In both cases, the most pronounced differences were observed between Group 1 and Group 4.

**Figure 4:**
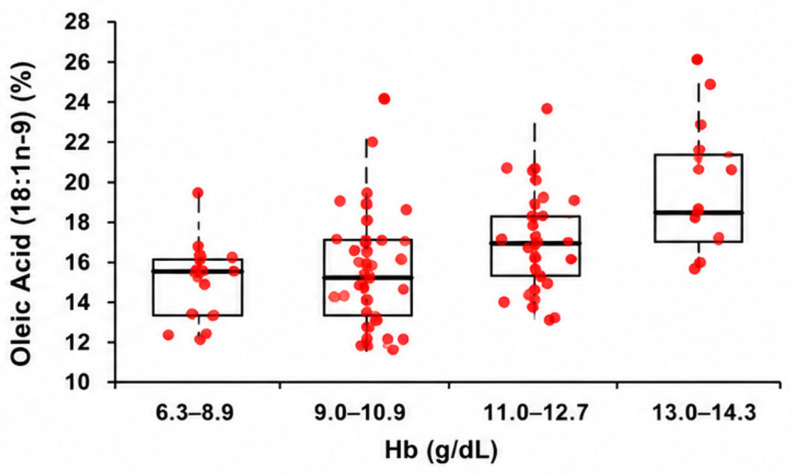
Distribution of oleic acid (18:1n-9) percentages according to different hemoglobin levels.

In terms of oleic acid levels, a statistically significant difference was observed between Hb groups (*p* < 0.0001), specifically between Group 1 and Group 2, Group 1 and Group 3, Group 1 and Group 4, and Group 2 and Group 3 (Figure 4).

In unadjusted analyses, Hb levels showed weak inverse associations with palmitic and erucic acid concentrations; however, these associations did not remain significant after false discovery rate (FDR) correction and should therefore be interpreted as exploratory. Conversely, elevated Hb levels were positively associated with oleic acid concentrations (r = 0.451, *p* < 0.0001), and this association remained significant after FDR correction, further supporting a potential relationship between monounsaturated fatty acids and erythropoietic activity.

**Figure 5:**
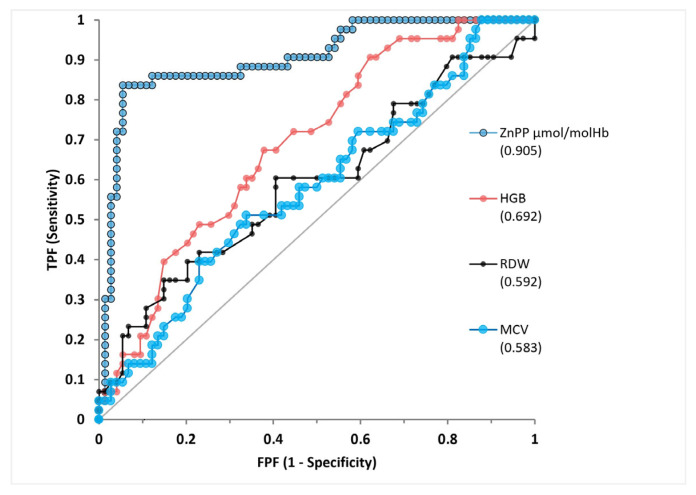
ROC curves showing the discriminatory performance of TSAT, ZnPP, Hb, RDW, MCV, and ferritin for identifying iron deficiency anemia in hemodialysis patients.

As a secondary analysis, receiver operating characteristic (ROC) curve analysis was performed to evaluate the discriminatory performance of ZnPP and conventional hematological and iron-related parameters for identifying IDA. ZnPP demonstrated the largest observed AUC in this cohort (0.905, 95% CI: 0.845–0.965, *p* < 0.0001), followed by TSAT (AUC 0.728), Hb (AUC 0.692), RDW (AUC 0.592), MCV (AUC 0.583), and ferritin (AUC 0.566) (Figure 5, Table 5). Pairwise comparison of ROC curves demonstrated that the AUC of ZnPP was significantly greater than those of ferritin (*p* < 0.0001), MCV (*p* < 0.0001), RDW (*p* < 0.0001), Hb (*p* < 0.0001), and TSAT (*p* = 0.006), indicating good discrimination within this study population.

The optimal ZnPP cut-off value was 13.1 μmol/molHb, corresponding to a sensitivity of 84% and a specificity of 95% (LR+ 15.5; LR− 0.2). The AUC values, 95% confidence intervals, standard errors, Z statistics, and corresponding *p*-values for all evaluated biomarkers are presented in Table 5.

**Figure 6:**
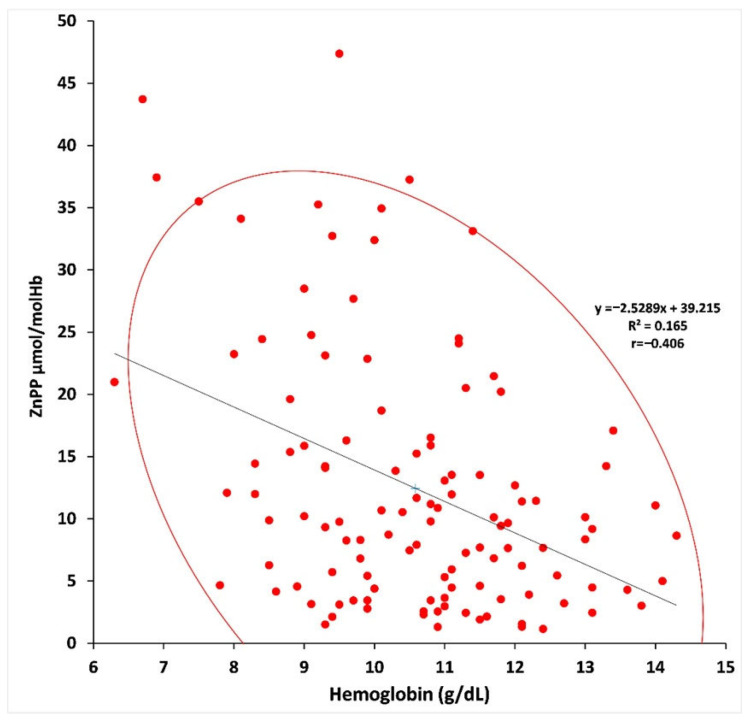
Correlation between ZnPP (μmol/molHb) and hemoglobin levels in hemodialysis patients.

A significant negative correlation was found between ZnPP and Hb in hemodialysis patients (r = −0.406, *p* < 0.0001) (Figure 6).

### 3.4. Multivariable Linear Regression Analysis of Hemoglobin Levels

To determine whether the associations observed in the univariate analyses remained independent after adjustment for potential confounding factors, a multivariable linear regression analysis was performed using hemoglobin concentration as the dependent variable. Age, sex, diabetes mellitus, CRP, iron supplementation, EPO use, ZnPP, palmitic acid, oleic acid, and erucic acid were included as covariates. The results of the multivariable model are presented in Table 6.

A multivariable linear regression analysis was performed using hemoglobin level as the dependent variable and adjusting for age, sex, diabetes mellitus, CRP, iron supplementation, and EPO use. Oleic acid (β = 0.167, 95% CI: 0.067–0.267, *p* = 0.001) and EPO use (β = 0.881, 95% CI: 0.310–1.451, *p* = 0.003) remained independently and positively associated with hemoglobin levels. In contrast, ZnPP remained independently and negatively associated with hemoglobin (β = −0.051, 95% CI: −0.076 to −0.026, *p* < 0.0001). Palmitic acid (*p* = 0.162) and erucic acid (*p* = 0.295), which were significant in univariate analyses, did not retain independent significance after adjustment for potential confounders (Table 6).

## 4. Discussion

In this study involving 117 HD patients and 28 healthy controls, we identified three principal findings: (i) HD patients exhibited a significantly reduced omega-3 index compared with healthy controls; (ii) Hb-defined anemia was characterized by elevated palmitic and erucic acid levels together with reduced oleic acid levels; and (iii) ZnPP levels differed significantly across hemoglobin-defined groups, with the highest values observed in patients with the lowest hemoglobin levels, and showed potential as a biomarker associated with iron-restricted erythropoiesis in HD patients. These findings suggest that alterations in erythrocyte membrane fatty acid composition and ZnPP metabolism may be associated with the pathophysiology of anemia in CKD.

Importantly, multivariable regression analysis demonstrated that the associations of oleic acid and ZnPP with hemoglobin levels remained significant after adjustment for age, sex, diabetes mellitus, CRP, iron supplementation, and EPO use. In contrast, palmitic acid and erucic acid lost statistical significance after adjustment, suggesting that these associations may be partly explained by clinical and treatment-related confounding factors. These findings were consistent with the FDR-adjusted correlation analyses, which identified oleic acid as the only fatty acid remaining significantly associated with hemoglobin after correction for multiple testing.

The positive association between EPO use and hemoglobin should be interpreted cautiously because of the cross-sectional design. Patients receiving EPO therapy may have achieved higher hemoglobin levels as a consequence of treatment response, and the observed association should not be interpreted as evidence of a causal effect.

CKD leads to systemic metabolic disturbances that impair EPO production, chronic inflammation, oxidative stress, and altered iron metabolism [1]. Anemia in CKD contributes to hypoxia-related vascular and renal dysregulation [16]. ZnPP levels were significantly higher in HD patients with lower hemoglobin levels (*p* = 0.013), likely reflecting impaired incorporation of iron into protoporphyrin during heme synthesis. Unlike ferritin, which may be influenced by chronic inflammation, ZnPP may provide a complementary indicator of iron-restricted erythropoiesis in HD patients [17]. RBC counts were markedly lower in HD patients with lower hemoglobin levels (*p* < 0.0001), supporting the impact of iron-restricted erythropoiesis and EPO insufficiency on hematologic parameters [17].

Hemoglobin, hematocrit, and serum iron were lower at lower hemoglobin levels, whereas ferritin was paradoxically higher and transferrin saturation remained relatively preserved. This combination of low hemoglobin with elevated ferritin and low-to-normal transferrin saturation is more consistent with iron-restricted erythropoiesis and with the KDIGO 2026 iron-treatment eligibility framework than with absolute (systemic) iron deficiency [2,18]. Because ferritin behaves as an acute-phase reactant and may remain elevated in HD patients owing to inflammation and altered iron handling, these findings should not be interpreted as evidence of depleted iron stores alone. Therefore, ferritin in CKD should be interpreted together with inflammatory markers and transferrin saturation [19]. HCT levels were also significantly reduced (*p* < 0.05), consistent with the suppressive effects of iron deficiency and reduced EPO on erythropoiesis [1,2].

Given these inflammatory limitations of ferritin, ZnPP may represent a clinically useful adjunctive biomarker for identifying iron-restricted erythropoiesis in HD patients. Although the primary analyses in this study were based on hemoglobin-defined groups to evaluate biomarker changes across the hemoglobin spectrum, a secondary ROC analysis was additionally performed using a predefined iron deficiency anemia (IDA) classification. In this analysis, IDA was operationally defined as anemia together with TSAT <20% and ferritin <200 ng/mL. ZnPP showed the largest observed AUC among the evaluated biomarkers (0.905, 95% CI: 0.845–0.965, *p* < 0.0001). Furthermore, pairwise ROC comparisons showed that the AUC of ZnPP was significantly greater than those of ferritin, MCV, RDW, Hb, and TSAT. These findings support the potential utility of ZnPP as a complementary marker reflecting iron-restricted erythropoiesis and suggest that ZnPP may provide additional information beyond conventional hematological and iron-related indices in hemodialysis patients. Alterations in erythrocyte membrane fatty acid composition represented another major finding of the study. Palmitic and erucic acids were elevated, whereas oleic acid was reduced in patients with anemia. Although palmitic and erucic acids showed inverse correlations with hemoglobin levels in unadjusted analyses, these associations did not remain significant after FDR correction. In contrast, the positive association between oleic acid and hemoglobin remained statistically significant after adjustment for multiple testing.

Decreased omega-3 fatty acids in HD patients may reflect chronic inflammation, oxidative stress, and metabolic dysregulation [20,21,22,23,24,25]. However, although omega-3 index levels were significantly lower in HD patients than in healthy controls, they did not differ significantly across hemoglobin-defined groups. These findings suggest that reduced omega-3 status may reflect generalized metabolic impairment in HD rather than an anemia-specific alteration.

Oleic acid may exert protective effects on erythrocyte membrane integrity and erythropoietic activity. Previous studies suggested that oleic acid may enhance erythropoiesis via transferrin receptor activation, improved membrane fluidity, and reduced oxidative injury [26,27,28]. Notably, oleic acid levels did not differ significantly between HD patients and healthy controls overall (Table 2, *p* = 0.482); however, oleic acid levels were significantly lower in HD patients with lower hemoglobin levels (Table 4, *p* < 0.0001). This pattern suggests that reduced oleic acid may represent an anemia-associated membrane alteration within the HD population and may reflect membrane remodeling accompanying increasing anemia severity, rather than a generalized CKD-associated change. Therefore, the positive association between oleic acid and Hb observed in the present study may indicate a compensatory or protective metabolic response in HD patients with preserved erythropoietic capacity.

In contrast, palmitic acid is known to induce inflammation, oxidative stress, mitochondrial dysfunction, and impaired iron metabolism [29,30,31,32]. Increased palmitic acid levels may therefore contribute to erythrocyte membrane instability and defective erythropoiesis in HD patients. Similarly, elevated erucic acid levels observed in HD patients with anemia may reflect CKD-associated dysregulation of very-long-chain fatty acid metabolism. Although erucic acid has been reported to exert some anti-inflammatory properties at low concentrations, its accumulation under chronic hypoxic and inflammatory conditions may impair mitochondrial beta-oxidation and disrupt membrane phospholipid remodeling [33,34,35]. These metabolic perturbations could compromise erythrocyte membrane integrity and contribute to reduced erythropoietic efficiency. However, the inverse association between erucic acid and hemoglobin observed in the unadjusted correlation analysis (r = −0.183, *p* = 0.048) did not remain statistically significant after Benjamini–Hochberg FDR correction and should therefore be interpreted as exploratory.

Increased monounsaturated fatty acids, alongside decreased EPA, DHA, and omega-3 index levels, indicate oxidative imbalance, membrane dysfunction, and malnutrition in HD patients [36,37]. Positive correlations between ZnPP and tridecanoic and behenic acids were observed in unadjusted analyses; however, these associations did not remain significant after FDR correction and should therefore be interpreted as exploratory [38,39]. These associations may indicate compensatory membrane-related responses during iron-restricted erythropoiesis. Collectively, these findings suggest that alterations in erythrocyte membrane fatty acid composition may contribute to anemia-related metabolic disturbances in HD patients.

It should also be noted that a recent randomized trial conducted in a community-based sub-cohort of the VITAL study found no significant reduction in anemia incidence following omega-3 FA supplementation over two years [40]. However, the VITAL trial was conducted in a general aging population without specific kidney disease, and whether these findings can be extrapolated to the chronic inflammatory and metabolically altered milieu of HD patients remains uncertain. Therefore, HD-specific randomized controlled trials investigating omega-3 supplementation are still required before definitive clinical recommendations can be made.

Several limitations of this study should be acknowledged. First, due to the cross-sectional design, causal relationships between erythrocyte membrane fatty acid alterations, ZnPP levels, and anemia-related parameters could not be established. In addition, the relatively short study period limited the evaluation of long-term biochemical and hematological changes.

Hepcidin and soluble transferrin receptor (sTfR) levels were not evaluated, which may have limited the comprehensive assessment of iron metabolism. Furthermore, clinically relevant biomarkers associated with iron metabolism and inflammation, including reticulocyte hemoglobin content (Ret-He/CHr), IL-6, TNF-α, and erythropoiesis-stimulating agent dose responsiveness, were not assessed. Ret-He/CHr measurements were not consistently available for all participants and were therefore not included in the final analysis. Inflammatory status was evaluated primarily using CRP levels, and more comprehensive inflammatory profiling was not performed.

In addition, 38.5% of patients were receiving iron supplementation and 64.1% were receiving erythropoiesis-stimulating agent therapy. Although these treatments were recorded and reported, their potential effects on hemoglobin levels, iron-related parameters, and observed associations could not be fully controlled because of the cross-sectional design of the study. Therefore, residual treatment-related confounding cannot be excluded.

Although multivariable regression analyses were performed, residual confounding cannot be completely excluded because of the observational cross-sectional design.

Furthermore, erythrocyte membrane fatty acids were expressed as relative percentages of total fatty acids and therefore represent compositional data. Consequently, correlations among individual fatty acids may occur and could contribute to multicollinearity in multivariable analyses. The regression findings should therefore be interpreted with appropriate caution.

Dietary intake, including fish consumption and omega-3 supplementation, as well as lipid-lowering medications such as statins and fibrates, were not systematically recorded during the study period and therefore could not be incorporated into the statistical analyses; consequently, these factors may have confounded erythrocyte membrane fatty acid measurements. Since erythrocyte membrane fatty acid profiles reflect long-term dietary habits, the absence of standardized dietary assessment—particularly regarding habitual fish consumption and omega-3 source intake—represents a relevant limitation that should be considered when interpreting membrane fatty acid data. In addition, the relatively small size of the control group (*n* = 28) compared with the patient group (*n* = 117) may have limited the statistical power of between-group comparisons involving healthy controls; future studies with larger, age- and sex-matched control populations are warranted. Furthermore, the significantly higher total cholesterol, HDL cholesterol, and LDL cholesterol levels observed in healthy controls compared with HD patients indicate that the two groups differed not only in renal status but also in baseline lipid profiles. Therefore, between-group comparisons involving lipid-related parameters should be interpreted with caution. Although healthy controls were included as a reference population, they may not fully reflect the complex metabolic, inflammatory, and lipid-related background of CKD or HD patients. Other metabolic characteristics, including dietary patterns, nutritional status, omega-3 intake, and lifestyle-related factors, were not systematically assessed and therefore could not be incorporated into the analyses.

Multiple correlation analyses were performed, and Benjamini–Hochberg FDR correction was applied to reduce the risk of type I error. Nevertheless, because a large number of exploratory analyses were conducted, some findings should still be interpreted cautiously and require validation in independent cohorts.

Furthermore, because erythrocyte membrane fatty acid percentages represent compositional data, correlations among individual lipid fractions may have introduced collinearity, which could have influenced the stability of multivariable regression estimates.

Future longitudinal, multicenter, and interventional studies, particularly those investigating omega-3 supplementation, iron metabolism biomarkers, and membrane-associated metabolic alterations, are warranted to validate and expand upon these findings.

## 5. Conclusions

To the best of our knowledge, this study is among the first to evaluate the combined relationship between erythrocyte membrane fatty acid composition, omega-3 index, and ZnPP levels across the spectrum of hemoglobin levels in HD patients. The findings demonstrate that HD patients with lower hemoglobin levels exhibit a distinct membrane lipid profile characterized by elevated palmitic and erucic acids and reduced oleic acid levels. Among the evaluated fatty acids, only the positive association between oleic acid and hemoglobin remained significant after FDR correction and multivariable adjustment. ZnPP levels differed significantly across hemoglobin-defined groups, with the highest values observed in patients with the lowest hemoglobin levels, and ZnPP was independently and inversely associated with hemoglobin after multivariable adjustment (β = −0.051, *p* < 0.0001). In ROC analysis, ZnPP demonstrated the highest discriminatory performance for identifying iron deficiency anemia, with an AUC of 0.905 (95% CI 0.845–0.965), significantly exceeding those of ferritin, MCV, RDW, Hb, and TSAT. Collectively, these findings suggest that membrane lipid profiling and ZnPP measurement may provide complementary information for the evaluation of anemia in hemodialysis patients. Future longitudinal and interventional studies are warranted to clarify causal relationships and evaluate the clinical utility of these biomarkers in routine HD practice.

## Figures and Tables

**Table 1:** Demographic and clinical characteristics of hemodialysis patients.

Characteristic (*n* = 117)	N	%
Gender		
Male	67	57.3
Female	50	42.7
Age Groups		
18–44	18	15.4
45–64	38	32.5
65–84	53	45.3
>85	8	6.8
Primary Disease, no, %		
Diabetes mellitus	35	29.9
Hypertension	66	56.4
Glomerulonephritis	2	1.7
Urological reasons	8	6.8
Polycystic kidney disease	9	7.7
Other	17	14.5
Dialysis time		
3–12 Months	20	17.1
13–60 Months	59	50.4
>61 Months	38	32.5
Smoking		
Yes	15	12.8
No	102	87.2
Alcohol use		
Yes	3	2.6
No	114	97.4
Vascular Access Information		
AV Fistula	63	53.8
Persistent Catheter	54	46.2
Iron Medication Use		
Yes	45	38.5
No	70	59.8
Unknown	2	1.7
EPO Use		
Yes	75	64.1
No	40	34.2
Unknown	2	1.7
ACE-I/ARB Use		
Yes	9	7.7
No	106	90.6
Unknown	2	1.7

Percentages may exceed 100% because some patients had multiple underlying causes of chronic kidney disease.

**Table 2:** A comparison of fatty acid levels in hemodialysis patients and control groups.

Erythrocyte Membrane Fatty Acids	Patients (*n* = 117)	Control (*n* = 28)	*p*-Value
Tridecanoic acid (%)	0.199 [0.112–0.326]	0.276 [0.082–0.513]	0.345
Myristic acid (%)	0.431 [0.336–0.599]	0.497 [0.309–0.632]	0.914
Pentadecanoic acid (%)	0.353 [0.255–0.432]	0.4 [0.242–0.71]	0.263
Palmitic acid (%)	27.861 [25.138–31.664]	25.142 [22.997–27.162]	0.001
Heptadecanoic acid (%)	0.369 [0.304–0.426]	0.363 [0.245–0.469]	0.824
Linoleic acid (%)	15.055 [13.692–16.476]	15.484 [13.974–17.617]	0.248
Oleic acid (%)	16.269 [14.407–17.994]	15.82 [14.213–17.074]	0.482
Stearic acid (%)	21.483 [19.047–23.91]	18.82 [17.213–20.074]	<0.001
Arachidonic acid (%)	12.671 [9.078–15.316]	13.726 [12.148–14.973]	0.119
Eicosapentaenoic acid (EPA) (%)	0.128 [0.073–0.308]	0.585 [0.343–1.02]	<0.0001
7,10,13 Eicosatrienoic acid (%)	1.043 [0.818–1.285]	1.191 [0.875–1.708]	0.069
Eicosanoic acid (%)	0.069 [0.043–0.115]	0.081 [0.056–0.121]	0.099
Docosahexaenoic acid (%)	1.869 [1.3–3.351]	2.721 [1.892–3.898]	0.010
CIS13,16-Docosadienoic acid (%)	0.012 [0.007–0.022]	0.011 [0.008–0.015]	0.672
Erucic acid (%)	0.053 [0.02–0.252]	0.034 [0.017–0.066]	0.030
Behenic acid (%)	0.189 [0.118–0.342]	0.321 [0.2–0.403]	0.045
Lignoceric acid (%)	0.035 [0.021–0.056]	0.039 [0.017–0.061]	0.875
Omega 3 index (%)	2.083 [1.463–3.659]	3.501 [2.717–4.397]	<0.0001
Total Cholesterol (mg/dL)	160 [130.6–183]	207 [178.75–230.25]	<0.0001
Triglyceride	146 [93.7–194]	140 [85–197.5]	0.839
HDL Cholesterol (mg/dL)	34.2 [24.2–43]	50.5 [43–56.25]	<0.0001
LDL Cholesterol (mg/dL)	85 [62.2–116]	123.5 [102.25–147.5]	<0.0001

Data are expressed as Median (25th–75th Percentile).

**Table 3:** Spearman correlation analysis between erythrocyte membrane fatty acids and biochemical parameters.

Erythrocyte Membrane Fatty Acids	WBC	RBC	Hb	RDW	MCV	ZnPP	TSAT	Iron	Ferritin	CRP	HCT	MCHC	PLT
Tridecanoic acid (%)	−0.005	−0.013	−0.037	0.078	−0.062	0.239	0.159	0.164	−0.059	−0.070	−0.035	−0.010	−0.009
Myristic acid (%)	−0.057	0.078	0.135	−0.078	0.205	0.002	−0.074	0.050	−0.063	−0.082	0.150	−0.071	0.009
Pentadecanoic acid (%)	0.043	−0.049	0.023	−0.103	0.251	0.071	0.022	0.113	0.002	−0.035	0.028	−0.012	0.053
Palmitic acid (%)	−0.159	−0.187	−0.220	−0.045	−0.023	0.102	−0.074	−0.082	0.033	−0.018	−0.197	−0.074	−0.110
Heptadecanoic acid (%)	−0.018	−0.086	0.022	−0.203	0.226	−0.031	0.088	0.042	0.076	−0.038	−0.012	0.084	0.143
Linoleic acid (%)	−0.127	0.111	0.130	0.093	−0.031	−0.047	−0.042	−0.049	−0.081	0.000	0.106	0.034	−0.065
Oleic acid (%)	−0.030	0.442 ***	0.451 ***	0.017	0.044	−0.150	−0.026	0.113	−0.037	−0.110	0.466 ***	−0.146	−0.086
Stearic acid (%)	0.121	−0.046	−0.013	−0.068	0.007	−0.029	−0.084	−0.046	−0.124	0.120	−0.050	0.127	0.038
Arachidonic acid (%)	0.129	−0.132	−0.207	0.032	−0.177	0.071	0.056	−0.027	0.044	−0.029	−0.192	0.030	0.133
Eicosapentaenoic acid (EPA) (%)	0.120	0.025	0.041	0.085	0.097	0.104	0.075	0.049	−0.037	−0.051	0.057	−0.050	0.144
7,10,13 Eicosatrienoic acid (%)	0.002	−0.191	−0.233	0.071	−0.068	0.139	−0.040	−0.098	0.021	−0.068	−0.214	−0.015	0.030
Eicosanoic acid (%)	0.097	−0.074	−0.071	0.078	−0.098	0.017	−0.126	−0.062	−0.136	0.087	−0.111	0.139	0.239
Docosahexaenoic acid (%)	0.058	−0.068	−0.071	0.021	−0.070	0.024	0.056	−0.033	0.019	0.021	−0.093	0.125	0.115
Cis-13,16-docosadienoic acid (%)	0.187	−0.090	−0.015	−0.050	0.148	−0.005	−0.144	−0.140	−0.044	0.106	−0.054	0.180	0.096
Erucic acid (%)	0.081	−0.226	−0.183	−0.051	0.002	0.007	−0.066	0.008	−0.043	0.116	−0.239	0.201	0.190
Behenic acid (%)	0.014	−0.071	−0.084	0.120	−0.050	0.184	0.158	0.124	−0.042	−0.051	−0.086	−0.014	0.016
Lignoceric acid (%)	0.102	−0.082	−0.093	−0.046	−0.134	0.101	−0.070	0.041	−0.123	0.096	−0.126	0.086	−0.009
Omega 3 index (%)	0.080	−0.057	−0.056	0.039	−0.042	0.046	0.069	−0.019	0.009	0.008	−0.072	0.103	0.138
Total Cholesterol (mg/dL)	0.093	0.003	0.088	−0.138	0.205	−0.007	−0.069	0.059	0.094	−0.027	0.066	0.056	0.172
Triglyceride (mg/dL)	0.121	0.077	0.127	−0.096	0.067	−0.013	−0.074	0.072	−0.036	−0.089	0.101	0.022	0.115
HDL Cholesterol (mg/dL)	−0.076	−0.161	−0.110	−0.003	0.180	0.109	0.056	−0.101	0.101	0.078	−0.119	0.100	0.064
LDL Cholesterol (mg/dL)	0.104	0.023	0.073	−0.122	0.174	−0.091	−0.080	0.066	0.112	−0.039	0.070	0.018	0.048

*** *p* < 0.001. q < 0.05 after Benjamini–Hochberg false discovery rate correction. Unadjusted Spearman correlation coefficients are shown. Associations that were significant before FDR correction but did not remain significant after correction were interpreted as exploratory.

**Table 4:** Laboratory Parameters According to Hemoglobin-Defined Groups.

Laboratory Parameters	Group 1 (*n* = 12)	Group 2 (*n* = 38)	Group 3 (*n* = 50)	Group 4 (*n* = 17)	*p*-Value
Hb (g/dL)	13.48 ± 0.44	11.65 ± 0.48	9.95 ± 0.60 *†	8.01 ± 0.74 *†‡	<0.0001
WBC (×10^9^/L)	6.285 [5.468–7.743]	6.47 [5.055–7.408]	5.92 [5.14–7.605]	5.79 [4.23–7.49]	0.487
RBC (×10^12^/L)	4.32 ± 0.34	3.82 ± 0.23	3.20 ± 0.37 *†	2.68 ± 0.25 *†‡	<0.0001
RDW (%)	15.68 ± 1.23	15.27 ± 1.18	15.29 ± 1.25	15.76 ± 0.97	0.204
MCV (fL)	94.30 ± 4.87	93.70 ± 4.25	94.29 ± 5.22	91.69 ± 5.20	0.473
ZnPP (μmol/molHb)	8.515 [4.449–10.38]	7.47 [3.723–12.516]	10.387 [4.665–16.481]	15.377 [9.893–24.452] ‡	0.013
TSAT (%)	34.09 [21.075–43.715]	31.485 [22.448–42.813]	33.49 [22.35–44.875]	30.56 [23.37–39.51]	0.912
Iron (µg/dL)	70.81 [63.75–75.28]	58 [44.125–78.018]	56.5 [42.075–73.875]	54.85 [41–67]	0.241
Ferritin (ng/mL)	413.61 [102.828–567.25]	624.5 [486.25–824.393]	753 [460.798–1029.25] *	798.09 [522–1203] *	0.017
CRP (mg/L)	3.32 [3.168–19.95]	8.3 [3.65–16.1]	8.7 [3.9–20.95]	10.1 [6.2–17]	0.630
HCT (%)	40.63 ± 1.92	35.72 ± 1.41	30.02 ± 2.77 *†	24.50 ± 2.24 *†‡	<0.0001
MCHC (g/dL)	32.65 [32.575–33.35]	32.55 [32.025–32.975]	32.8 [32.225–33.5]	32.8 [32.5–33.1]	0.439
PLT (×10^9^/L)	214.16 ± 52.78	199.89 ± 65.33	187.78 ± 61.67	201.64 ± 88.24	0.510
Albumin (g/L)	39.85 [38.505–41.28]	38.25 [35.725–39.845]	37.5 [35.25–39.608]	35.3 [32.2–37.8] *	0.010
Urea (mg/dL)	112.29 [99.375–119.048]	112.8 [96.25–131.5]	107.5 [91.125–127.323]	94 [87.5–106.6]	0.123
Creatinine (mg/dL)	8.72 ± 2.41	6.99 ± 2.43	7.33 ± 2.20	7.16 ± 3.44	0.140
Tridecanoic acid (%)	0.242 [0.126–0.411]	0.201 [0.097–0.334]	0.21 [0.134–0.303]	0.15 [0.07–0.214]	0.516
Myristic acid (%)	0.417 [0.339–0.483]	0.538 [0.398–0.749]	0.401 [0.331–0.559]	0.396 [0.282–0.541]	0.067
Pentadecanoic acid (%)	0.267 [0.196–0.378]	0.387 [0.321–0.486]	0.354 [0.301–0.419]	0.276 [0.242–0.404]	0.053
Palmitic acid (%)	25.397 [24.443–25.717]	28.059 [25.702–32.12]	27.645 [24.756–31.022]	30.691 [27.575–31.888] *	0.016
Heptadecanoic acid (%)	0.35 ± 0.06	0.37 ± 0.10	0.36 ± 0.12	0.36 ± 0.09	0.794
Linoleic acid (%)	15.88 ± 3.43	15.64 ± 2.73	14.96 ± 2.67	15.10 ± 2.19	0.635
Oleic acid (%)	19.81 ± 2.88 ◊ • ▪	16.92 ± 2.00	15.52 ± 2.61 *†	15.23 ± 1.52 *	<0.0001
Stearic acid (%)	20.70 ± 3.45	21.47 ± 3.29	21.53 ± 3.43	20.91 ± 2.81	0.753
Arachidonic acid (%)	11.611 [8.876–15.894]	11.023 [8.611–14.107]	13.663 [10.437–15.554]	14.428 [9.223–18.315]	0.150
Eicosapentaenoic acid (EPA) (%)	0.092 [0.053–0.156]	0.127 [0.076–0.312]	0.135 [0.073–0.206]	0.129 [0.102–0.313]	0.753
7,10,13 Eicosatrienoic acid (%)	1.008 [0.835–1.187]	0.866 [0.772–1.139]	1.097 [0.864–1.385]	1.137 [0.747–1.285]	0.075
Eicosanoic acid (%)	0.061 [0.035–0.085]	0.067 [0.05–0.107]	0.073 [0.043–0.131]	0.069 [0.041–0.104]	0.633
Docosahexaenoic acid (DHA) (%)	1.63 [1.277–3.66]	1.785 [1.208–2.376]	1.971 [1.322–3.396]	2.294 [1.639–3.726]	0.594
CIS13,16-Docosadienoic acid (%)	0.008 [0–0.017]	0.011 [0.008–0.021]	0.015 [0.009–0.023]	0.01 [0.006–0.019]	0.144
Erucic acid (%)	0.016 [0.007–0.041]	0.042 [0.019–0.259]	0.044 [0.024–0.254] *	0.174 [0.071–0.312] *	0.004
Behenic acid (%)	0.137 [0.098–0.203]	0.211 [0.113–0.342]	0.19 [0.12–0.408]	0.233 [0.153–0.342]	0.455
Lignoceric acid (%)	0.041 [0.027–0.054]	0.066 [0.048–0.078]	0.072 [0.045–0.1]	0.044 [0.033–0.103]	0.171
Omega-3 index (%)	1.672 [1.350–3.758]	1.957 [1.475–2.589]	2.138 [1.660–3.759]	2.383 [1.767–4.157]	0.589
Total Cholesterol (mg/dL)	156.49 ± 36.82	161.15 ± 36.55	160.25 ± 45.26	154.72 ± 43.81	0.591
Triglyceride (mg/dL)	160.90 ± 66.66	163.84 ± 90.62	155.00 ± 117.72	129.85 ± 53.43	0.948
HDL Cholesterol (mg/dL)	33.90 ± 12.47	30.64 ± 15.08	34.76 ± 14.41	35.57 ± 16.70	0.356
LDL Cholesterol (mg/dL)	76.33 ± 30.88	94.54 ± 34.24	90.45 ± 39.41	82.39 ± 41.34	0.505

Data are expressed as mean ± standard deviation (SD) or median (interquartile range, IQR), as appropriate. Data are presented as mean ± SD or median [IQR]. Comparisons among groups were performed using one-way ANOVA with Tukey post hoc testing for normally distributed variables and Kruskal–Wallis analysis with Bonferroni-adjusted pairwise comparisons for non-normally distributed variables. For Kruskal–Wallis post hoc comparisons: * *p* < 0.05 vs. Group 1; † *p* < 0.05 vs. Group 2; ‡ *p* < 0.05 vs. Group 3. For ANOVA post hoc comparisons: ◊ *p* < 0.05 vs. Group 2; • *p* < 0.05 vs. Group 3; ▪ *p* < 0.05 vs. Group 4.

**Table 5:** ROC curve analysis of biomarkers for identifying iron deficiency anemia in hemodialysis patients.

Biomarker	AUC	95% CI	SE	Z Statistic	*p*-Value
Transferrin Saturation (%)	0.728	0.627–0.830	0.0518	4.41	<0.0001
ZnPP (μmol/molHb)	0.905	0.845–0.965	0.0307	13.18	<0.0001
Hemoglobin (g/dL)	0.692	0.596–0.788	0.0491	3.91	<0.0001
RDW (%)	0.592	0.481–0.704	0.0569	1.62	0.1044
MCV (fL)	0.583	0.475–0.691	0.0551	1.50	0.1331
Ferritin (ng/mL)	0.566	0.456–0.677	0.0564	1.18	0.2399

IDA was operationally defined as anemia together with TSAT < 20% and ferritin < 200 ng/mL. ROC analysis was performed in 43 patients with IDA and 74 patients without IDA. AUC values are presented with corresponding 95% confidence intervals (CI). Cut-off values were determined using the optimal diagnostic threshold derived from ROC curve analysis. Sensitivity, specificity, positive likelihood ratio (LR+), and negative likelihood ratio (LR−) are reported for each biomarker. Because ferritin and TSAT were incorporated into the operational definition of IDA, the ROC estimates for these biomarkers should be interpreted descriptively and may be affected by incorporation bias.

**Table 6:** Multivariable linear regression analysis of factors independently associated with hemoglobin levels in hemodialysis patients.

Variable	β	95% CI	*p*-Value
Age	0.010	−0.005 to 0.025	0.182
ZnPP (μmol/molHb)	−0.051	−0.076 to −0.026	<0.0001
CRP (mg/L)	−0.002	−0.008 to 0.005	0.644
Palmitic acid (%)	−0.041	−0.098 to 0.017	0.162
Oleic acid (%)	0.167	0.067 to 0.267	0.001
Erucic acid (%)	−0.944	−2.723 to 0.834	0.295
Sex	−0.186	−0.696 to 0.323	0.470
Diabetes mellitus	0.004	−0.540 to 0.549	0.987
Iron supplementation	0.008	−0.558 to 0.574	0.979
EPO use	0.881	0.310 to 1.451	0.003

## Data Availability

The original contributions presented in this study are included in the article. Further inquiries can be directed to the corresponding author.

## Abbreviations

The following abbreviations are used in this manuscript:

ACE-I	Angiotensin-converting enzyme inhibitors
ANOVA	Analysis of variance
ARB	Angiotensin II receptor blockers
AUC	Area under the curve
BHT	Butylated hydroxytoluene
BMI	Body mass index
CHr	Reticulocyte hemoglobin content
CI	Confidence interval
CKD	Chronic kidney disease
CRP	C-reactive protein
DHA	Docosahexaenoic acid
DMSO	Dimethyl sulfoxide
EPA	Eicosapentaenoic acid
EPO	Erythropoietin
FAMEs	Fatty acid methyl esters
GC-MS	Gas chromatography–mass spectrometry
Hb	Hemoglobin
HCT	Hematocrit
HD	Hemodialysis
HDL	High-density lipoprotein
HPLC	High-performance liquid chromatography
IDA	Iron deficiency anemia
IL-6	Interleukin-6
IQR	Interquartile range
LDL	Low-density lipoprotein
LOD	Limit of detection
MCHC	Mean corpuscular hemoglobin concentration
MCV	Mean corpuscular volume
MUFA	Monounsaturated fatty acid
PLT	Platelet count
PUFA	Polyunsaturated fatty acid
RBC	Red blood cell count
RDW	Red cell distribution width
Ret-He	Reticulocyte hemoglobin equivalent
ROC	Receiver operating characteristic
SD	Standard deviation
SFA	Saturated fatty acid
sTfR	Soluble transferrin receptor
TNF-α	Tumor necrosis factor-alpha
TSAT	Transferrin saturation
WBC	White blood cell count
ZnPP	Zinc protoporphyrin
